# Phosphorylation and Activation of the Plasma Membrane Na^+^/H^+^ Exchanger (NHE1) during Osmotic Cell Shrinkage

**DOI:** 10.1371/journal.pone.0029210

**Published:** 2011-12-28

**Authors:** Robert R. Rigor, Catalina Damoc, Brett S. Phinney, Peter M. Cala

**Affiliations:** 1 Department of Physiology and Membrane Biology, School of Medicine, University of California Davis, Davis, California, United States of America; 2 Proteomics Core Facility, Genome Center, University of California Davis, Davis, California, United States of America; University of Pennsylvannia, United States of America

## Abstract

The Na^+^/H ^+^ Exchanger isoform 1 (NHE1) is a highly versatile, broadly distributed and precisely controlled transport protein that mediates volume and pH regulation in most cell types. NHE1 phosphorylation contributes to Na^+^/H^+^ exchange activity in response to phorbol esters, growth factors or protein phosphatase inhibitors, but has not been observed during activation by osmotic cell shrinkage (OCS). We examined the role of NHE1 phosphorylation during activation by OCS, using an ideal model system, the *Amphiuma tridactylum* red blood cell (atRBC). Na^+^/H^+^ exchange in atRBCs is mediated by an NHE1 homolog (atNHE1) that is 79% identical to human NHE1 at the amino acid level. NHE1 activity in atRBCs is exceptionally robust in that transport activity can increase more than 2 orders of magnitude from rest to full activation. Michaelis-Menten transport kinetics indicates that either OCS or treatment with the phosphatase inhibitor calyculin-A (CLA) increase Na^+^ transport capacity without affecting transport affinity (K_m_ = 44 mM) in atRBCs. CLA and OCS act non-additively to activate atNHE1, indicating convergent, phosphorylation-dependent signaling in atNHE1 activation. *In situ* ^32^P labeling and immunoprecipitation demonstrates that the net phosphorylation of atNHE1 is increased 4-fold during OCS coinciding with a more than 2-order increase in Na^+^ transport activity. This is the first reported evidence of increased NHE1 phosphorylation during OCS in any vertebrate cell type. Finally, liquid chromatography and mass spectrometry (LC-MS/MS) analysis of atNHE1 immunoprecipitated from atRBC membranes reveals 9 phosphorylated serine/threonine residues, suggesting that activation of atNHE1 involves multiple phosphorylation and/or dephosphorylation events.

## Introduction

The type 1 Na^+^/H^+^ exchanger (NHE1) is a ubiquitously distributed plasma membrane protein that is centrally involved in many physiological processes including fluid secretion, apoptosis, cell growth/proliferation, cell pH regulation and cell volume regulation (for review see [1], [2], [3], [4], [5], [6], [7]. NHE1 mediates cell volume regulation in response to osmotic cell shrinkage (OCS) in a process known as regulatory volume increase (RVI). Appropriate activation of NHE1 during RVI depends upon signaling mechanisms that are very poorly understood [2], [3], [5], [7], [8], [9], [10], [11], [12], [13]. In general, control of NHE1 transport activity involves multiple intracellular signaling molecules, including MAP/ERK kinases and Ca^++^/calmodulin, as well as phosphorylation (or dephosphorylation) at one or more serine (S) residues of the cytosolic C-terminus of NHE1, including (human numbering) S648, S703, S723, S726, S729, S770, S771, S785, and S796 [14], [15], [16], [17], [18], [19], [20], [21], [22]. Phosphorylation of NHE1 *in situ* in living cells occurs in response to treatment with growth factors, okadaic acid, phorbol esters, sustained intracellular acidification or angiotensin II [15], [18], [23], [24], [25], [26]. However, phosphorylation of NHE1 has never been demonstrated in response to OCS [23], [27], [28], suggesting that OCS increases NHE1 transport activity through a mechanism that differs from other forms of activation.

Although net phosphorylation of NHE1 is not increased during OCS in mammalian cells, it is possible that failure to observe increased NHE1 phosphorylation is due to the modest increases in NHE1 activity that are typical of the volume regulation response in mammalian cells [29]. Because patterns of NHE1 phosphorylation are complex in response to various stimuli, increases in phosphorylation of NHE1 may be masked by simultaneous dephosphorylation at separate serine loci within the C-terminal cytosolic domain of NHE1 [23]. We reasoned that modest increases in net phosphorylation of NHE1, if present, would be more readily observed in a cell type with more robust inducible NHE1 activity, the *Amphiuma tridactylum* RBC (*at*RBC).

The *at*RBC is an ideal model for the study of Na^+^/H^+^ exchange activity during RVI [13], [30]. In contrast to mammalian cells, NHE1 transport activity is virtually nonexistent in quiescent atRBCs. Following suspension in hyperosmotic media, *at*RBCs exhibit an increase in NHE1-mediated Na^+^ flux activity that is nominally 2-orders of magnitude greater than that of cells at normal resting volume in isosmotic medium [31], [32], [33]. In addition, the *at*RBC NHE1 homolog (*at*NHE1) is 79% identical to human NHE1 at the amino acid level, retains the hallmark housekeeping characteristics of mammalian NHE1: cell pH and volume regulation, and is expressed in abundance in *at*RBCs compared to cell types known to over-express NHE1 (e.g., tumor cells) [33], [34]. Therefore the atRBC is an excellent model in which to study both physiological and biochemical basis for NHE1 activation.

Recently we proposed a model in which OCS activates NHE1 via a rate-limiting phosphorylation-dependent signal transduction event consisting of forward (activating) kinase activity that is activated by OCS, and reverse (inactivating) phosphatase activity that is inactivated by OCS [32]. This model is based on analysis of relaxation kinetics describing activation and inactivation of NHE1 transport activity, in response to acute osmotic shrinkage or re-swelling after OCS, respectively. The notion that phosphorylation is involved in control of NHE1 activity during OCS is based on the observation that NHE1 inactivation is prevented under various osmotic conditions by treatment with the protein phosphatase (PP1/PP2A) inhibitor calyculin-A (CLA) [31]. These functional studies provide a detailed kinetic description of the rate-limiting biochemical events in NHE1 activation and inactivation during osmotic cell volume perturbation, and while strongly suggestive of a phosphorylation-dependent mechanism, do not firmly establish a role for phosphorylation in NHE1 activation by OCS, or that CLA treatment affects the same cell signaling events as OCS.

In the present study, we use the atRBC model to provide kinetic and biochemical evidence that activation of NHE1 during OCS involves phosphoryation-dependent signaling as well as direct phosphorylation of the NHE1 protein. Na^+^ transport kinetics confirms that OCS and CLA increase NHE1 activity using an identical biochemical mechanism, and through convergent upstream signaling. Because CLA is a phosphatase inhibitor, this implies that phosphorylation is involved in these processes. We further examine the net phosphorylation status of the NHE1 protein directly with ^32^P-orthophosphate labeling, and provide the first reported evidence that NHE1 is phosphorylated *in situ* in response to OCS. Candidate sites of phosphorylation or dephosphorylation are subsequently identified using NHE1 immunoprecipitated from atRBCs and mass spectrometry (LC-MS/MS). These studies confirm that phosphorylation is involved in activation of NHE1 during OCS, and demonstrate the complexity of NHE1 phosphorylation *in situ* in living cells.

## Results

### Na^+^ transport kinetics of Na+/H+ exchange in osmotically shrunken cells

We previously demonstrated that activation of NHE1-mediated Na^+^ transport activity in osmotically shrunken atRBCs is dependent upon a rate-limiting phosphorylation-dependent biochemical event [31]. The behavior of this rate-limiting event is consistent with that of a simple kinase and phosphatase pair, where the NHE1-inactivating phosphatase activity is inhibited by treatment with the protein phosphatase inhibitor CLA. The activities of both the activating kinase and the inactivating phosphatase are cell volume-dependent. In unstimulated cells at normal volume, phosphatase activity is dominant and maintains NHE1 in a tonic inactivated state. NHE1-inactivating phosphatase activity decreases precipitously with cell shrinkage upon suspension of cells in hyperosmotic media [32]. In contrast, NHE1-activating kinase activity increases as a graded function of cell shrinkage in increasingly hyperosmotic media, imparting exquisite volume sensitivity to Na^+^/H^+^ exchange activity. Because the rate-limiting event in shrinkage-activation of NHE1 is sensitive to the phosphatase inhibitor CLA, a major implication of the model is that this rate-limiting event involves protein phosphorylation. Furthermore, OCS and CLA treatment are presumed to affect identical downstream phosphorylation-dependent events in the activation of NHE1. Thus, it follows that the endpoint biochemical mechanisms controlling NHE1 activity are identical in response to OCS or CLA treatment. To test the notion that OCS and CLA treatment utilize the same biochemical mechanisms to increase NHE1 activity, we examined NHE1 activity, including Michaelis-Menten Na^+^ transport kinetics in osmotically shrunken atRBCs.

**Figure 1:**
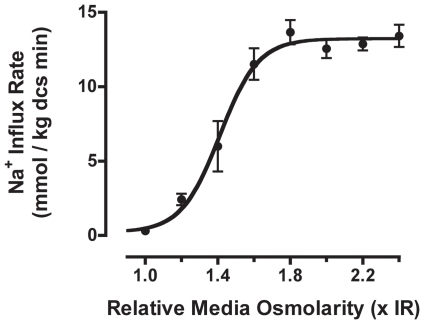
The stimulus-response relationship between medium osmolarity and NHE1 Na^+^ transport activity in atRBCs. Unidirectional Na^+^ influx rates (^22^Na^+^ initial rates) were measured in hyperosmotic media at a fixed media [Na^+^] = 100 mM, following complete activation of NHE1 by pre-incubation in thermodynamically nulled media of matched osmolarity. The data are fit to a sigmoidal curve by non-linear regression (Hill slope = 4.1), yielding a maximal Na^+^ influx rate of 13.2 mmoles Na^+^ kg^−1^ dcs minute. (data are n≥5 each; ± SE).

First, we assessed the shrinkage-dependent Na^+^ transport activity by NHE1 in atRBCs suspended in hyperosmotic media. Na^+^ transport activity was determined following complete activation of NHE1 by pre-incubation in thermodynamically nulled (n) media (with respect to Na^+^/H^+^ exchange) of matched hyperosmolarity, thereby clamping RBCs at their initial shrunken volumes prior to flux determinations (described previously [31], [32]). Briefly, in nulled solutions, low medium Na^+^ concentration prohibits net Na^+^ uptake and precludes cell volume recovery, allowing Na^+^/H^+^ exchange to become fully activated and to remain in the activated state. NHE1 activity was then assessed by tracer ^22^Na^+^ uptake in media of fixed Na^+^ concentration (100 mM), to determine initial Na^+^ influx rates over a broad range of media osmolarities. These Na^+^ influx rate data conformed well to a sigmoidal stimulus-response relationship between media osmolarity and Na^+^ flux activity (Figure 1), though with a steep Hill coefficient (n_H_ = 4) reflecting cooperativity in the cell volume-dependent signal transduction. The data demonstrate a more than 2-order of magnitude increase in inducible Na^+^/H^+^ exchange activity (180-fold) (maximal Na^+^ influx rate = 13.23±0.43 mmoles Na^+^ kg dcs^−1^ minute^−1^) relative to basal Na^+^ flux rates for NHE1 in unstimulated RBCs in isosmotic medium (0.073±0.041 mmoles Na^+^ kg dcs^−1^ minute^−1^ (n = 9; ±SEM)).

### CLA does not affect Na^+^ transport activity of NHE1 following maximal shrinkage-activation

**Figure 2:**
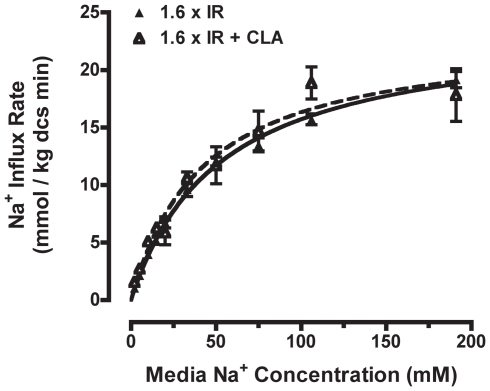
Na^+^ transport kinetics of NHE1 in hyperosmotic medium. Michaelis-Menten Na^+^ transport kinetics were determined for NHE1 in *at*RBCs following complete activation in hyperosmotic (1.6×IR) media, with (closed triangles) or without (open triangles) 500 nM CLA (data are compiled from n≥3 independent experiments; means ± SE). The data are fit by regression to a simple hyperbola, and the corresponding kinetic constants are reported in Table 1. The Na^+^ transport kinetics (activity) are virtually identical in the two conditions.

Next we determined if OCS and CLA activate NHE1 through independent or convergent activation mechanisms. In the most extreme example of divergence, CLA and OCS might activate separate pools of NHE1 within the cell through entirely independent signaling mechanisms. We reasoned that if the stimuli affect separate populations of NHE1 then the effects of CLA and OCS should be strictly additive with respect to NHE1 activity, regardless of the extent of NHE1 activation by OCS. To assess this more precisely, we examined the Michaelis-Menten Na^+^ transport kinetics of NHE1 in atRBCs following induction of maximal NHE1 activity in hyperosmotic medium with or without CLA treatment. Based on the stimulus-response information in Figure 1, NHE1 reached maximum activity following suspension of RBCs in hyperosmotic (nulled 1.6×IR) medium. K_m_ and J_max_ were then determined with or without CLA present (Figure 2). K_m_ values were similar with or without CLA (K_m_ = 42.0±8.4 or 52.1±3.9 mM Na^+^, respectively) (Table 1). In addition, the J_max_ values for maximally shrinkage-stimulated NHE1 were virtually identical with or without CLA treatment (J_max_ = 23.2±1.8 or 23.9±0.8 mmoles Na^+^ kg dcs^−1^ minute^−1^, respectively) (Table 1). Therefore, under conditions of maximal NHE1 activation by OCS, CLA treatment does not further activate NHE1. This indicates that OCS and CLA treatment activate the same population of NHE1, and suggests that OCS and CLA treatment activate NHE1 through convergent signaling.

**Table 1:** Na^+^ transport kinetic constants for NHE1 in *Amphiuma* RBCs maximally stimulated to steady-state in hyperosmotic (1.6×IR) media with or without 500 nM CLA treatment.

Treatment	K_m_(mM Na^+^)	J_max_ (mmoles Na^+^/Kg dcs minute)
Hyperosmotic (1.6×IR)	52.1±3.9	23.9±0.8
Hyperosmotic (1.6×IR) + CLA	42.0±8.4	23.2±1.8

Kinetic constants are expressed as K_m_ or J_max_ ± SEM. Values for K_m_ and J_max_ are virtually identical in both treatment conditions.

### Osmotic cell shrinkage and calyculin-A treatment synergistically activate NHE1

**Figure 3:**
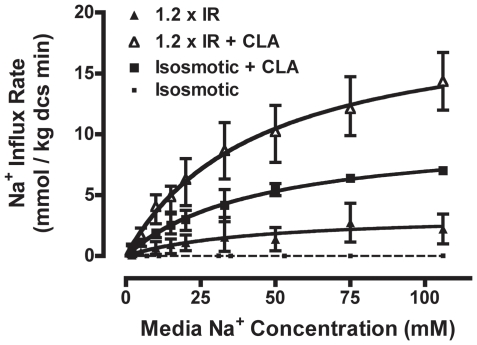
Na^+^ transport kinetics of NHE1 in mildly hyperosmotic medium. The Michaelis-Menten Na^+^ transport kinetics were determined for NHE1 in *at*RBCs following complete activation in mildly hyperosmotic (1.2×IR) medium alone (closed triangles), hyperosmotic (1.2×IR) medium with 500 nM CLA (open triangles), or CLA treatment alone in IR (squares). Data for resting atRBCs in isosmotic medium are shown for comparison (small squares, dashed curve). The data are fit by regression to a simple hyperbola, and the corresponding kinetic constants are reported in Table 2. The Na^+^ transport affinity is not significantly different across the three conditions. However, the maximal transport rate J_max_ is significantly increased in hyperosmotic media together with CLA treatment, relative to either treatment alone (p<0.05). (data are compiled from n≥3 independent experiments; means ± SE).

Although CLA and OCS clearly activate the same population of NHE1 protein, these data cannot rule out the possibility that NHE1 is activated through separate upstream signaling events terminating in a common mechanism of NHE1 activation (e.g., increased surface expression, etc.), such that NHE1 cannot be further activated once in the maximally activated state. If this were true, then the two treatments would be strictly additive under conditions of modest NHE1 activation [35], [36]. In contrast, if effects on NHE1 activity are not strictly additive, this indicates convergence of the signaling pathways upstream of NHE1 activation. To thoroughly assess this relationship, we suspended atRBCs in mildly hyperosmotic (1.2×IR) medium, with or without CLA treatment, and compared NHE1 activity (Michaelis-Menten Na^+^ transport kinetics) to that in response to CLA treatment alone in isosmotic medium (Figure 3). As seen before, basal NHE1 activity in isosmotic medium is virtually non-existent (estimated J_max_ = 0.02±0.01 mmoles Na^+^/kg^−1^ dcs min^−1^; K_m_ could not be reliably determined in the unstimulated state). Consistent with the transport kinetics of NHE1 in the maximally activated state, the K_m_ values for NHE1 were identical (K_m_≅44 mM) in all three stimulated conditions (Table 2). In contrast, CLA treatment and OCS were supra-additive with respect to NHE1 activity, in that the J_max_ for NHE1 in osmotically shrunken RBCs in the presence of CLA (19.8±2.5 mmoles Na^+^ kg^−1^ dcs min^−1^) was significantly greater than the sum of the individual mean J_max_ values for osmotic cell shrinkage and CLA stimulation (13.8 mmoles Na^+^ kg^−1^ dcs min^−1^) (p<0.05) (Figure 3; Table 2). These data are consistent with the notion that CLA and OCS activate NHE1 via convergence on identical upstream signaling events. Because CLA is a phosphatase inhibitor, these data suggest that shrinkage activation of NHE1 is phosphorylation-dependent, however it remains unclear whether activation involves direct phosphorylation of NHE1.

**Table 2:** Na^+^ transport kinetic constants for NHE1 in *Amphiuma* RBCs stimulated to steady-state in isosmotic solution with 500 nM CLA, or mildly hyperosmotic (1.2×IR) solution ± CLA.

Treatment	Km(mM Na+)	Jmax (mmoles Na+/Kg dcs minute
Isosmotic (1×IR)	NA	0.02±0.01*
Isosmotic (1×IR) + CLA	44.3±7.7	10.4±1.4
Hyperosmotic (1.2×IR)	43.3±36.8	3.4±1.4
Hyperosmotic (1.2×IR) + CLA	44.4±11.8	19.8±2.5

Kinetic constants are expressed as K_m_ or J_max_ ± SEM. Values for K_m_ are similar in all 3 conditions, while J_max_ is increased by CLA treatment relative to hyperosmotic medium alone. Estimated* (by hyperbolic curve-fit) J_max_ activity in the unstimulated isosmotic condition is also shown.

### NHE1 phosphorylation during osmotic cell shrinkage

**Figure 4:**
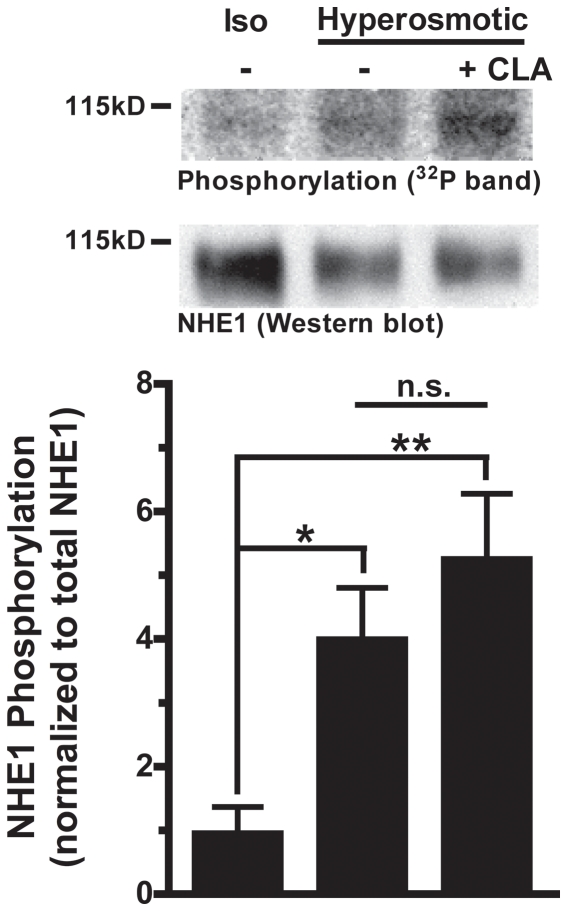
*In situ* [^32^P]-orthophosphate labeling of NHE1 during cell suspension in hyperosmotic medium. (upper) ^32^P incorporation into immunoprecipitated NHE1 from *at*RBCs. autoradiograph images corresponding to immunoprecipitated NHE1 from membrane fractions of [^32^P]-orthophosphate labeled *at*RBCs suspended in isosmotic medium, or hyperosmotic media (n1.6×IR) with or without CLA treatment. Below each autoradiograph band is the corresponding NHE1 Western blot band detected on the same PVDF membrane. (lower) A quantitative comparison of data from the autoradiograph bands described in panel A (normalized to NHE1 Western blots from the same PVDF membrane). Relative phosphorylation of immunoprecipitated NHE1 is significantly increased by suspension of cells in hyperosmotic (n1.6×IR) solution with or without CLA treatment relative to the isosmotic control (*p<0.05 , **p<0.01; n = 6±SEM). No significant difference is detected between the two treatment conditions (hyperosmotic ± CLA; n.s.).

To determine if NHE1 activation involves direct NHE1 phosphorylation, we assessed the net phosphorylation status of NHE1 by *in situ* [^32^P]-orthophosphate labeling. RBCs were pre-equilibrated with [^32^P]-orthophosphate and then exposed to isosmotic, or hyperosmotic medium (1.6×IR) with or without CLA treatment (the same treatment conditions as in Figure 2). ^32^P-labeled NHE1 was then immunoprecipitated from RBC membranes, and analyzed by autoradiography to quantify ^32^P incorporation into NHE1. Net phosphorylation of NHE1 was increased 4-fold (4.0±1.9; ± SE; n = 6) by suspension of atRBCs in hyperosmotic medium alone, and 5-fold (5.3±2.4; ± SE; n = 6) with inclusion of CLA. These values were not significantly different from each other (p>0.05), however they were significantly increased relative to baseline in isosmotic medium (*p<0.05, **p<0.01; 1.0±0.9; ± SE; n = 6) (Figure 4). Notably, earlier experiments suggested that treatment with CLA in isosmotic medium alone yields a 2-fold increase in NHE1 phosphorylation with this treatment compared to isosmotic medium in the absence of CLA (2.0±0.3 (mean ± SE); n = 3; p = 0.1). Therefore, NHE1 is phosphorylated during OCS. These phosphorylation results are also consistent with the Na^+^ transport activity data in Figure 2 showing no further effect of CLA exposure for cells where NHE1 is maximally activated in extreme hyperosmotic (1.6×IR) medium. However there is an enormous difference between the more than 2-order increase in Na^+^ transport activity and the corresponding net increase in NHE1 phosphorylation (4-fold) during OCS, suggesting that the molecular mechanism of NHE1 activation is more complex than that of a simple unimolecular phosphorylation event.

### Phosphorylation sites on NHE1 identified by mass spectrometry

**Figure 5:**
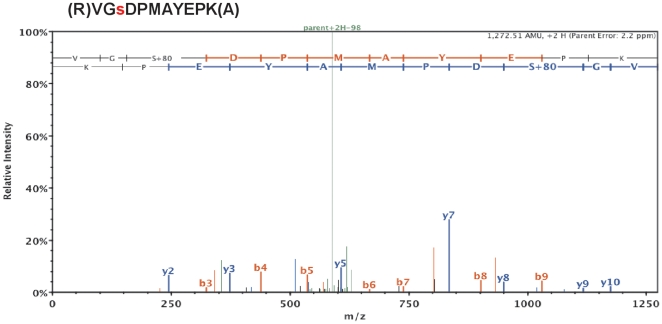
Ion Trap MS/MS spectrum of a fragmented peptide containing phosphorylated serine 711 of *at*NHE1. NHE1 was immunoprecipitated from atRBC membranes, SDS-PAGE purified and in-gel digested with trypsin. The resulting peptides were extracted an analyzed by LC-MS/MS. MS/MS spectra were generated by collision-induced dissociation of individual peptides, which preferentially fragments peptides at peptide bonds to generate N-terminal (b ions) and C-terminal (y ions) fragments with characteristic charge/mass (m/z) ratios identifying the amino acid composition of the collection of fragments. In this representative spectrum, the (2+) y ions are labeled in red and positioned above the b ions labeled in blue. Phosphorylation of serine 711 is shown as a gain of 80-kDa corresponding to H_3_PO_4_. MS/MS spectra identifying other sites of NHE1 phosphorylation are on file in the Tranche database at ProteomeCommons.org (see Methods).

The discrepancy between the amount of net NHE1 phosphorylation and the magnitude of inducible NHE1 activity strongly suggests that NHE1 activation involves synergistic effects of multiple phosphorylation sites, including the possibility of simultaneous phosphorylation and dephosphorylation of NHE1. To identify exact sites of NHE1 phosphorylation *in situ*, we qualitatively examined the post-translational modifications of NHE1 by liquid chromatography (LC) and tandem mass spectrometry (MS/MS). The immunoprecipitation products of three in situ treatments were examined: IR, 1.6×IR, and CLA treatment (as described for Figures 2 and 3). NHE1 was identified (100% probability) in all samples by amino acid sequence data comprising ≥26% coverage of the NHE1 protein, including >60% coverage of the cytosolic C-terminal amino acids, and multiple post-translational modifications. Analysis of y and b ions in MS^2^ spectra from tryptic peptides identified 9 phosphorylated serine/threonine sites: with greater than 90% probability (Figure 5; Table 3; Complete spectra are on file at ProteomeCommons.org). Phosphorylated peptides containing the following phosphorylated sites were found in all samples (*Amphiuma* numbering): S607, S610, S711, and S783. Four additional sites were identified from phosphorylated peptides found only in the 1.6×IR, and CLA treatments, but not in the IR (unstimulated) condition: S613, T693, T727, and S794. The phosphorylation site S701 was also identified, though only in one sample due to lack of coverage of this residue in the MS/MS data. A table summarizing the qualitative identification of phosphorylated peptide fragments is included as Table S1. Together, the data support the conclusion that NHE1 is phosphorylated on multiple residues of the cytosolic C-terminus, and that the phosphorylation pattern is similar in response to OCS or CLA treatment.

**Table 3:** NHE1 phosphorylation sites detected *in situ* in *at*RBCs by LC-MS/MS.

Phospho-site,*Amphiuma* NHE1	Peptide detected,*Amphiuma* NHE1	Corresponding sites,human NHE1
S607, S610	^605^IP**s**AV**s**TVSMQNIQPK^620^	S599, S602
S613	^605^IPSAVSTV**s**MQNIQPK^620^	S605
T693, S701	^688^MNNYL**t**VPAHKMDsPTMTR^706^	T685, S693
S711	^709^VG**s**DPMAYEPK^719^	S703
T727	^724^DLP**t**ITIDPASPESVDIVNEEKK^746^	V716
S783	^766^EPPSPGTDDVFTPGAGD**s**PNNQR^788^	S785
S794	^792^CL**s**DPGPQPEPEEQDPFIKK^811^	S796

Phosphorylation sites (lower case, bold) detected from immunoprecipitated *Amphiuma* NHE1 are listed along with the specific tryptic peptide identified by LC-MS/MS, and the corresponding amino acid loci in the human NHE1 sequence (right column).

## Discussion

### Biochemical mechanisms and control of NHE1 activity during OCS or CLA treatment

Control of volume regulatory inorganic ion transport activity in response to cell volume disturbances depends upon fundamental cell physiological processes that are very poorly understood. The biochemical and molecular events involved in OCS-induced NHE1 activation are completely unknown. In the present study, we first described NHE1 transport activity using enzyme kinetics as a basis for understanding upstream cell signaling and the putative role of phosphorylation in OCS-induced NHE1 activation. We found that two treatments, OCS and CLA, increase NHE1 activity via the same biochemical mechanism: increased maximal Na^+^ transport rate (J_max_), with no effect on Na^+^ transport affinity (K_m_). Several laboratories, including ours, have noted that NHE1 activity can also be increased through increased Na^+^ transport affinity, e.g., in response to decreased intracellular pH [33]. The Na^+^ transport kinetics for NHE1 in the present study (Figures 2, 3; Tables 1, 2) support our earlier studies of NHE1 in *at*RBCs in hyperosmotic media, at physiological pH in and out [33]: the apparent K_m_ for Na^+^ is approximately 45 mM (at normal physiological pH_in_ = 7.1), and Na^+^ transport activity is manifest entirely as an increase in J_max_, and further demonstrate that these transport kinetic properties are the same in response to CLA treatment. Therefore the biochemical mode of NHE1 activation is the same whether induced by OCS or CLA treatment, yet fundamentally differs from that of intracellular acidification. Because CLA is a protein phosphatase inhibitor this further implies that activation of NHE1 by OCS is phosphorylation-dependent, however other possible mechanisms may exist to explain this behavior.

Previously we proposed a model for control of NHE1 activity in which NHE1 is activated by OCS or CLA through effects on a single rate-limiting activation event consisting of an activating kinase and an opposing phosphatase [31], [32]. Although our kinetic and functional analyses in the present study fully support this model, the assumption that phosphorylation is involved in OCS-induced signaling is based solely on the observation that CLA treatment activates NHE1, coupled with the fact that CLA is a protein phosphatase inhibitor. To further validate the volume-sensitive kinase/phosphatase model it was necessary to demonstrate unequivocally that CLA and OCS activate NHE1 through the same biochemical mechanisms and upstream cell signaling events. In the present study, we observed that CLA treatment and OCS are supra-additive with respect to NHE1 activity under conditions of modest OCS (Fig. 3), yet there is no additional activity elicited by CLA treatment during maximal shrinkage activation of NHE1 (Fig. 2). Therefore identical cell signaling events are affected by these treatments with respect to NHE1 activation. While this is not absolute evidence, these data strongly support the conclusion that NHE1 activation by OCS involves protein phosphorylation. However the notion that activation involves direct phosphorylation of the NHE1 protein remained to be tested.

### Phosphorylation of NHE1 during osmotic cell shrinkage

In an earlier study of NHE1 phosphorylation in cultured mammalian cells [23], Grinstein observed no increase in NHE1 phosphorylation in cells exposed to hyperosmotic medium, and speculated that the apparent lack of change in net phosphorylation with osmotic cell shrinkage reflects simultaneous phosphorylation and dephosphorylation at separate amino acid loci. Upon investigation of the phosphorylation status of NHE1 in atRBCs by *in situ* ^32^P labeling, we observed that the net phosphorylation of NHE1 is increased 4-fold during OCS compared to unstimulated cells in isosmotic medium (Figure 4). This is in contrast to a more than 2-order of magnitude increase in transport activity (Figure 1). This difference in phosphorylation versus activity suggests that NHE1 activity is increased through synergistic biochemical events involving NHE1 phosphorylation, and possibly including simultaneous dephosphorylation at separate amino acid loci. Because of the assumption that NHE1 is phosphorylated exclusively on serine residues, we initially probed for phosphorylation by immunoprecipitating NHE1 from atRBC membrane fractions followed by Western blotting with an anti-phosphoserine directed antibody (as described previously for *P. americanus* NHE1 [27]). Using this approach, we observed basal phospho-serine reactivity in the band corresponding to NHE1, yet no change in intensity of reactivity when RBCs were treated either with CLA or suspension in hyperosmotic solution (data not shown; p>0.05; n≥5). However, detection using phospho-serine antibodies is less sensitive than ^32^P labeling and is limited by the types of epitopes that can be recognized. In this case the phospho-serine antibody (4A3; Biomol International, LP, Plymouth Meeting, PA, USA) specifically detects phosphorylated serine residues adjacent to positive or uncharged amino acid residues, therefore it is possible that many phospho-sites are not detected. This is very likely the case in that 50% of the phospho-serine sites identified in Table 3 are adjacent to negatively charged residues.

**Figure 6:**
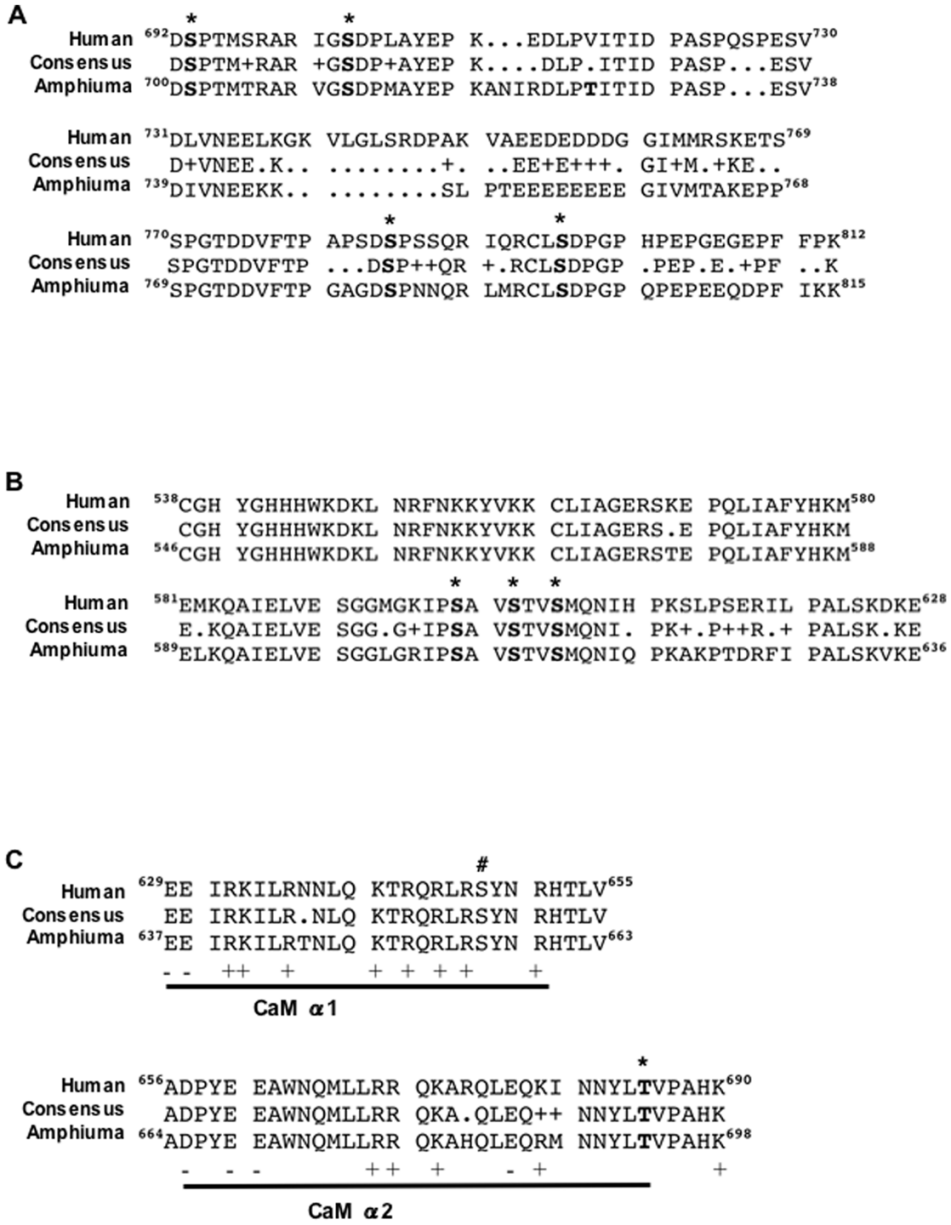
Comparison of C-terminal amino acid consensus sequence and phosphorylation sites in human NHE1 versus *Amphiuma* NHE1. Human NHE1, Amphiuma NHE1 and consensus sequences are shown for regions of the cytosolic C-terminus that contain phosphorylation sites identified by LC-MS/MS. A. The distal C-terminus. Shown in bold with asterisks are the locations of 4 conserved phosphorylated serine residues within this region: (Amphiuma) S701, S711, S783 and S794. T727, a site unique to Amphiuma NHE1, is also shown in bold. Residues with similarity are noted with + in the consensus sequence. B. The proximal C-terminus. Three conserved phosphorylation sites within this region are shown in bold with asterisks: (Amphiuma) S607, S610 and S613. C. Comparison of sequence information and phosphorylation sites of the volume-sensitive calmodulin (CaM) binding region in human versus *Amphiuma* NHE1. The helical domains necessary for CaM binding shown (α1 and α2) are based on structural studies by Köster et al [42], indicating CaM binding domains that are necessary for cell shrinkage-induced transport activity [41]. This region is 97% identical between human and *Amphiuma* NHE1. Shown in bold with an asterisk is the location of (Amphiuma) threonine 693, a conserved phosphorylation site within this region. The location of human S648 is also noted (#). Positive and negatively charged residues within the CaM binding regions are denoted below with + or −.

Because in most studies increased NHE1 phosphorylation is observed exclusively on serine residues distal to amino acid 698 of the cytosolic C-terminus (Figure 6A), we suspect that the same is true for increased phosphorylation of NHE1 during OCS. Within this region, three sites: S703 (S711), S785 (S783) and S796 (S794) are detected by LC-MS/MS as phosphorylated residues *in situ* in atRBCs. These loci correspond to sites where increased phosphorylation is associated with increased activity in mammalian cells: phosphorylation at S703 by the p90 ribosomal S6 kinase (p90RSK) is necessary for NHE1 activity in response to growth factor treatment [22] or angiotensin II [21]; S785 (S783) is phosphorylated in vitro by ERK2 [20]; and phosphorylation of S796 (S794) by ERK2 is required for the regulated association of carbonic anhydrase II with the distal C-terminus of NHE1 [19]. Because our LC-MS/MS analysis of phosphorylation was performed in a qualitative fashion, it is not possible to determine whether phosphorylation is increased or decreased. However, phosphorylation of one of these sites (S796) was identified only in precipitates from OCS- or CLA- treated cells, and not from unstimulated (IR) cells. This suggests that increased phosphorylation of residues S711, S783 and/or S796 contributes to increased NHE1 phosphorylation during activation in response to OCS. The specific contributions of these sites to NHE1 activity remains to be tested through systematic site-directed mutagenesis, and activity assays in heterologous expression systems.

Potentially other phosphorylation sites contribute to control of NHE1 activity in atRBCs. Our LC-MS/MS data identifies phosphorylation of three serine residues not previously reported, S599 (S607), S602 (S610), and S605 (S613) within the proximal C-terminus of NHE1 (Figure 6B). It is not clear if this region plays a role in the NHE1 response to OCS, however, this region of NHE1 is near to the binding domain for Nck-interacting kinase (NIK) which binds between residues 538–638 of NHE1 [37]. NIK also phosphorylates NHE1 (putatively at S648) in response to platelet-derived growth factor (PDGF) [14], [37], [38]. Speculatively, phosphorylation of residues S599/S602/S605 might interfere with or enhance NHE1 binding to NIK or to other proteins that are involved in the cellular response to OCS such as ezrin/radixin/moeisin (ERM) proteins [39].

### A putative role for decreased phosphorylation of NHE1 in activation during OCS

Because the increase in NHE1 activity during OCS is disproportionately greater than the measured increase in net NHE1 phosphorylation, it is equally likely that other sites exist where simultaneous decreases in phosphorylation occur during OCS. In mammalian cells, few examples exist in which increased NHE1 activity coincides with dephosphorylation of NHE1. Studies of truncated NHE1 mutants expressed in mammalian fibroblasts have shown that phosphorylation of NHE1 is decreased in the region of residues 635–698 in response to serum treatment [40]. This region is also shown to be essential for NHE1 activity in response to hyperosmolarity [29]. Within this region of NHE1 (Figure 6B), phosphorylation of (human) serine 648 is shown to be inhibitory toward NHE1 activity in rat ventricular myocytes [17]. However, mutation of S648 to alanine (S648A) fails to inhibit increased NHE1 activity in response to hyperosmotic medium [14], which is more consistent with the interpretation that dephosphorylation of S648 is permissive of NHE1 activation in response to OCS.

Dynamic interactions of S648 with calmodulin (CaM) may explain the role of dephosphorylation in activation of NHE1. In mammalian cells calmodulin (CaM) binding is necessary for OCS-induced NHE1 activity [41]. Studies of NHE1 and calmodulin (CaM) demonstrate that NHE1 exists in an autoinhibited state, and that CaM binding to residues 637–691 (Figure 6C) releases NHE1 from autoinhibition. It is further shown that phosphorylation of S648 prevents CaM binding and prevents NHE1 activation [17]. Recent X-ray scattering studies of the CaM binding regions of NHE1 reveal that phosphorylation of S648 disrupts CaM binding to helix α1 of the high affinity CaM binding domain of NHE1 [42]. This is accomplished in part by the electrostatic attraction of phosphorylated S648 for neighboring NHE1 arginine residues (R647 and R651), which in turn prevents salt bridge formation between NHE1 and CaM. According to this model, S648 dephosphorylation is necessary for CaM binding to NHE1. Structural modeling indicates that other NHE1 arginine (R632, R643) and glutamine (Q640, Q644) residues in this domain are involved in binding to CaM. Thus, dephosphorylation of NHE1 within the CaM binding domain may be a general prerequisite for NHE1 activation. This notion is supported by earlier studies showing that replacement of positively charged residues with negatively charged residues (mimicking phosphorylation) at various locations within the CaM binding domain substantially reduces NHE1 activity in response to OCS [41], [43]. Our current LC-MS/MS data did not include coverage of residues corresponding to 635–651 (human), however phosphorylation was detected at T693 (human T685) within the intermediate affinity CaM binding domain. We made preliminary attempts to examine the role of this site in site-directed mutagenesis studies with the human NHE1. Human NHE1 constructs were created and expressed in an NHE1-deficient cell line (AP-1) with T685 mutated to alanine (T685A) or aspartate (T685D). Cell Na^+^ uptake was significantly higher in the T685A mutant cell line compared to T685D in response to hypertonicity (p<0.05 at 90 minutes; analyzed by Two-way ANOVA with a Bonferroni post-test for significance) suggesting that the presence of a negative charge at T685 decreases the response of NHE1 to hypertonicity (unpublished data by RR Rigor). Further studies of this kind are necessary to more precisely determine the role of phosphorylation/dephosphorylation within the CaM binding domains in control of NHE1 activity during OCS.

### Conclusion

The evidence presented here support a role for phosphorylation in the cell signaling mechanisms upstream of NHE1 activation, and that NHE1 is directly phosphorylated during OCS. NHE1 activation by OCS involves phosphorylation of the distal C-terminus of NHE1, coinciding with predicted dephosphorylation of the CaM binding domains at residues 635–698. However the specific contribution of each phosphorylated or dephosphorylated locus must be further tested by systematic site-directed mutagenesis and biochemical analysis of the C-terminal serine residues of NHE1. The complex requirements for activation by OCS can readily explain why changes in net phosphorylation of NHE1 were not previously seen in studies with mammalian cells. In contrast, NHE1 phosphorylation is clearly seen during OCS in atRBCs due to the robust volume regulatory responses of this model system. This information is important for the fundamental understanding of cell volume regulation in confirming that phosphorylation-dependent signaling and direct phosphorylation of NHE1 are integral to OCS-induced NHE1 activation.

## Materials and Methods

This work was approved by the University of California Davis Animal Welfare Assurance on file with the US Public Health Service, under the IACUC approved animal care and use protocol no. 07-12754.

### Materials

^22^Na^+^ was obtained from New England Nuclear (NEN; Perkin Elmer, Waltham, MA) as NaCl; ^32^P-orthophosphate (500 µCi/µl) was from MP Biomedicals (Irvine, CA); reagent grade chemicals, dimethyl pimelimidate (DMP), ouabain and 5,5′ N-ethylisopropyl-amiloride (EIPA) were from Sigma-Aldrich (St. Louis, MO); Mini Complete protease inhibitor tablets were from Roche Diagnostics (Indianapolis, IN); kinase inhibitors and phosphatase inhibitors were from Calbiochem (EMD Biosciences, Gibbstown, NJ); protein G sepharose fast-flow beads were from Amersham (GE Healthcare, Piscataway, NJ); Anti-phosphoserine mouse monoclonal antibody (4A3) was from Biomol (Enzo Life Sciences, Farmingdale, NY); Anti-NHE1 mouse monoclonal antibody (MAb3140) was from Chemicon (Millipore, Billerica, MA); X-ray film was from Eastman Kodak; Narrow 500 µl PE microcentrifuge tubes were from E&K Scientific (Santa Clara, CA); Heparin (1000 U/ml) was from Henry Schein (Melville, NY).

### Physiological solutions

HEPES buffered Ringer's solutions: (mM) KCl 3, MgCl_2_ 1.0, CaCl_2_ 0.5, HEPES 30, NaOH 18, glucose 5, pH 7.65±0.02 were adjusted for desired osmolarity with NaCl, or NMDGCl to either reduce or fix solution [Na^+^]. The osmolarity of Isosmotic Ringer's (IR) solution, is 240±3 mOsm. The osmolarities of hyperosmotic solutions were adjusted relative to normal physiological osmolarity (240 mOsm) and are listed as a multiple of Isosmotic Ringer, i.e. 1.2, 1.4, 1.6… × IR. Thermodynamically nulled (n) flux media were formulated by adjusting media K^+^ and Na^+^ concentrations such that the net thermodynamic driving force for volume regulatory ion transporters (K^+^/H^+^ exchange or Na^+^/H^+^ exchange) is set equal to zero, as described previously [31], [32], [44]. Solutions used for Michaelis-Menten Na^+^ transport kinetics were formulated by varying media Na^+^ as described previously [33]. All solutions were gassed with humidified room air for 3 minutes at room temperature and pH adjusted to 7.65±0.02 just prior to initiation of the experiments. All flux media contained 1 mM ouabain to inhibit Na^+^/K^+^-ATPase activity.

### Preparation of erythrocytes

Wild captured adult *Amphiuma tridactylum* were purchased from Atchafalaya Biological Supply (Raceland, LA) and maintained in filtered freshwater tanks. Blood was drawn by cardiac puncture, into heparinized (1000 U) 20 ml syringes. Serum osmolarity for animals used in this study ranged 240±20 mOsm, as measured with an Advanced Instruments (Norwood, MA) model 3D3 freezing point depression osmometer. RBCs were washed three times by low-speed centrifugation (1500 g, 1 minute), supernatant was removed by vacuum aspiration, and the RBC pellet was resuspended in IR solution for 60 minutes at room temperature (22°C) to permit cells to reach steady-state, then stored overnight (4°C) for use the next day. Prior to experimentation, cell suspensions were adjusted to room temperature for 30 minutes in fresh IR solution. All RBC experiments were performed at 10% hematocrit (hct).

### Na^+^ transport activity assays

Na^+^ transport activity was assayed as the unidirectional influx of ^22^Na^+^ [31], [32], [33]. Briefly, RBCs were incubated in flux media containing ^22^Na^+^ (2 µCi/ml), and sampled over a brief interval (2–5 minutes) to measure initial flux rate and minimize backflux. Aliquots of RBC suspension were added to isotope-free ‘cold’ flux medium layered over dibutyl phthalate, and separated by centrifugation (1 min, 15,000 g). RBC pellets were isolated by inverting the 1.5 ml tubes and cutting off the conical tips. Radioactivity in the cell pellets were quantified with a Packard gamma counter, and normalized to the amount of dried cell solids. Prior to flux measurements, RBCs were pre-incubated in nulled (n) hyperosmotic media to allow for complete activation of Na^+^/H^+^ exchange, as described previously [32]. In all experiments, NHE1-mediated Na^+^ flux was operationally defined as EIPA-sensitive Na^+^ influx.

### *In situ* phosphorylation

RBCs were treated in a similar fashion to that described by Lytle [45], and Musch et al [46]. Briefly, *Amphiuma* RBCs were incubated to steady-state on a tube rotator in IR solution containing 500 µCi/ml [^32^P]-orthophosphate (10% hct, 4°C, 18 hours). Labeled RBCs were equilibrated to room temp (30 min), then sub-divided into equal volumes (0.25 ml/sample) and transferred to nIR or n1.6×IR solutions in the continued presence of [^32^P]-orthophosphate for 1 hour (with or without CLA in the latter 45 minutes, where indicated). Following the incubation period, RBCs were centrifuged (1 min, 5 k×g) and RBC pellets were flash frozen in liquid N_2_ for 10 minutes.

### Immunoprecipitation

Frozen RBC pellets were resuspended in (4°C) lysis buffer (PBS (pH 7.4) plus (mM) sodium fluoride 30, tetrasodium pyrophosphate 20, EDTA 5, EGTA 2, orthovanadate 1, ß-glycerophosphate 40, dithiothreitol 1, and Mini Complete protease inhibitor (Roche) (1 tab/10 ml). Lysates were centrifuged (15 k×g, 1 minute, 4°C), and the resulting pellets were washed twice more in 20 volumes of lysis buffer (4°C). Washed pellets were suspended in immunoprecipitation (IP) buffer (lysis buffer with 0.5% triton ×100) (4°C), incubated for 20 minutes on ice, and centrifuged (4°C, 15,000 g, 10 minutes). Supernatants were used for IP. IP beads were prepared by covalently crosslinking anti-NHE1 antibody to protein G sepharose beads by treatment with dimethyl pimelimidate (DMP), essentially as described by Schneider et al [47]. 25 µl of protein G beads were incubated with 10 µg of antibody in 0.1 M boric acid buffer (pH 8.2), on a rotator for 30 mins at room temp. Beads were washed twice in borate buffer, and twice in triethanolamine (0.2 M, pH 8.2), followed by DMP (18 mg/ml in triethanolamine) on a rotator for 45 minutes. The reaction was quenched with ethanolamine (70 mM, pH 8.2), then washed in borate buffer, PBS, and stored in IP buffer at 4°C. Sample supernatants were pre-cleared by incubation with DMP-treated beads (no antibody) for 30 minutes, and the resulting supernatants were added to the antibody-linked beads (4°C, rotating overnight). The following day, the beads were washed gently in IP buffer and NHE1 was harvested by addition of SDS-PAGE sample buffer. Harvested samples were run by SDS-PAGE (Tris-Glycine 7.5% Ready Gels; Bio-Rad Laboratories, Hercules, CA) and transferred to PVDF membranes. Membranes bearing ^32^P labeled bands were incubated with a phosphor-imaging screen for 5 days for autoradiography using a STORM Imager (Molecular Devices). PVDF membranes were subsequently rehydrated for Western blotting and chemiluminescence. Integrated band intensities were quantified using NIH Image J software. Background corrected ^32^P band intensities were normalized to total phosphorylation. Corresponding NHE1 bands detected by Western blotting were normalized to total NHE1 and used to express relative NHE1 phosphorylation (^32^P/unit NHE1). The calculated IP efficiency was 52% (n = 5). A similar IP protocol with IP materials scaled up 10-fold was used to prepare samples for mass spectrometry, starting with 0.5 ml of cell pellet material. 5% of the harvested material was used to confirm successful IP of NHE1 by Western blot. The remaining 95% was separated on large format SDS-PAGE gels (7.5%), stained with Colloidal Coomassie Blue stain (Novex) to confirm purity, and the NHE1 band was excised for protein extraction and trypsin digest prior to mass spectrometry, carried out as described previously [48], [49].

### NanoLC-MS/MS analysis

NanoLC-MS/MS experiments were performed on a Finnigan LTQ-FT hybrid linear ion trap/7T Fourier transform ion cyclotron resonance mass spectrometer (Thermo Electron, San Jose, CA, USA), equipped with a Finnigan Nanospray ion source (Thermo Electron), a Finnigan Surveyor MS pump (Thermo Electron), and a Finnigan micro-autosampler (Thermo Electron). The tryptic peptide mixture was separated on a 50 µm ID PicoFrit column packed in-house with Magic C18AQ material (Michrom BioResources, Inc., Auburn, CA, USA). The column was packed to a length of 12 cm with a 100% MeOH slurry of C18 reversed-phase material (100A pore size, 3 µm particle size) using a high-pressure cell pressurized with helium. The column was equilibrated before sample injection for 10 min at 2% solvent B (0.1% (v/v) formic acid in acetonitrile) and 98% solvent A (0.1% (v/v) formic acid in water) at a flow rate of 140 nl/min. Separation was achieved by using a linear gradient from 2 to 50% solvent B in 24 min at a flow rate of 320 nl/min. The LTQ-FT mass spectrometer was operated in the data dependent acquisition mode using the TOP10 method: a full-scan MS acquired in the FTICR mass spectrometer was followed by 10 MS/MS experiments performed with the LTQ on the ten most abundant ions detected in the full-scan MS.

### Database searching

Tandem mass spectra were extracted and charge state deconvoluted by bioworks version 3.3. Deisotoping was not performed. All MS/MS samples were analyzed using X! Tandem (The GPM, thegpm.org; version TORNADO (2010.01.01.4)). X! Tandem was set up to search the uniprot Amphibian database including an equal number of reverse sequences (release 2010_09, 108124 entries) and 101 contaminant protein sequences from the common Repository of Adventitious Proteins, cRAP database (thegpm.org/crap) assuming the digestion enzyme trypsin. X! Tandem was searched with a fragment ion mass tolerance of 0.40 Da and a parent ion tolerance of 20 PPM. Iodoacetamide derivative of cysteine was specified in X! Tandem as a fixed modification. Deamidation of asparagine and glutamine, oxidation of methionine and tryptophan, sulphone of methionine, tryptophan oxidation to formylkynurenin of tryptophan, acetylation of the n-terminus and phosphorylation of serine, threonine and tyrosine were specified in X! Tandem as variable modifications. The LC-MS/MS data associated with this manuscript may be downloaded from ProteomeCommons.org Tranche network using the following hash: tKUCAKV1XQpg3fiEuLGer5jUKXooAjuhpnDBqQTtpfuDIFwKKYPj1lhhL6hQuYuyFApkn9g/Mn3b9c6IMJ4NvfP32wcAAAAAAAAHIQ =  = .

### Criteria for protein identification

Scaffold (version Scaffold_3_00_04, Proteome Software Inc., Portland, OR) was used to validate MS/MS based peptide and protein identifications. Peptide identifications were accepted if they could be established at greater than 80.0% probability as specified by the Peptide Prophet algorithm [50]. Protein identifications were accepted if they could be established at greater than 99.0% probability and contained at least 1 identified peptides. This corresponded to a calculated protein and peptide FDR of 0.0% (Decoy/Target as discussed in JPR 2008 p45-6) Protein probabilities were assigned by the Protein Prophet algorithm [51]. Proteins that contained similar peptides and could not be differentiated based on MS/MS analysis alone were grouped to satisfy the principles of parsimony.

### Data regression and statistical analysis

General data analysis was performed with Prism 4.0 (Graph Pad) software. Stimulus-response data were fit to a sigmoidal function by non-linear regression. For transport kinetics, data were pooled from several identical experiments and fit to a single-binding site hyperbola by non-linear regression to generate values for K_m_ and J_max_ ± SEM. Tests for significance (p<0.05) were performed using Student's t-test with a two-tailed distribution.

## Supporting Information

Table S1 **A summary of LC-MS/MS data generated from analysis of atNHE1.** This complete list of NHE1 phosphorylated peptides identified by LC-MS/MS includes other post-translational modifications, as well as probability of identification, X!Tandem values, mass:charge (m/z), mass, charge and mass confidence (delta PPM) information.
(TIF)
