# Supplementary figures and images for: Phosphorylation and Activation of the Plasma Membrane Na+/H+ Exchanger (NHE1) during Osmotic Cell Shrinkage
Source: PLoS One. 2011 Dec 28;6(12):e29210. doi: 10.1371/journal.pone.0029210 (PMC3247252; doi:10.1371/journal.pone.0029210)

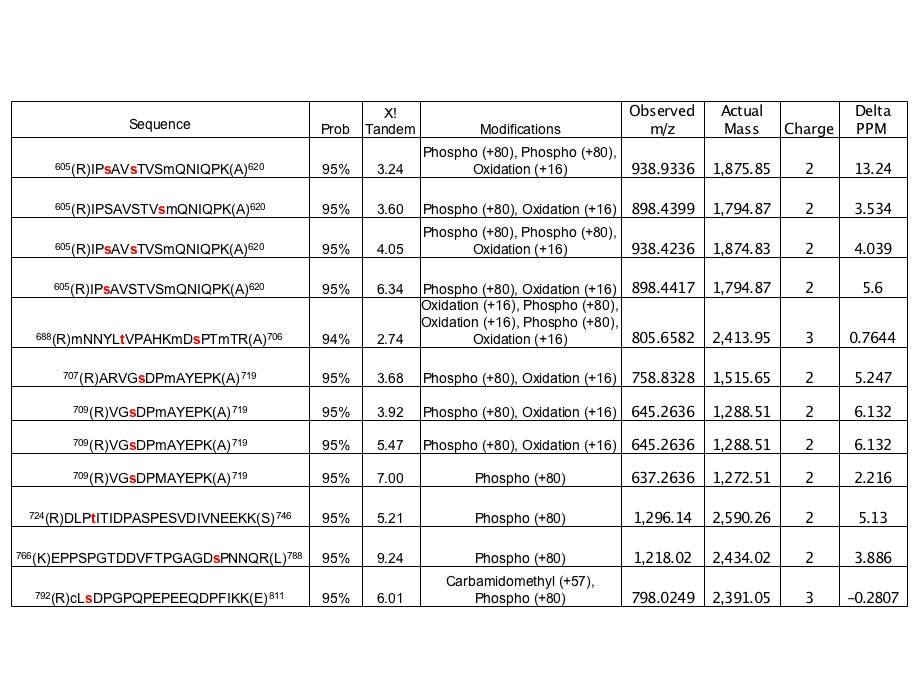

Supplement: Table S1 — A summary of LC-MS/MS data generated from analysis of atNHE1. This complete list of NHE1 phosphorylated peptides identified by LC-MS/MS includes other post-translational modifications, as well as probability of identification, X!Tandem values, mass:charge (m/z), mass, charge and mass confidence (delta PPM) information. (TIF) [file pone.0029210.s001.tif]
